# Cognitive Decline Is Associated with Risk Aversion and Temporal Discounting in Older Adults without Dementia

**DOI:** 10.1371/journal.pone.0121900

**Published:** 2015-04-02

**Authors:** Bryan D. James, Patricia A. Boyle, Lei Yu, S. Duke Han, David A. Bennett

**Affiliations:** 1 Rush Alzheimer’s Disease Center, Chicago, IL, United States of America; 2 Department of Internal Medicine, Rush University Medical Center, Chicago, IL, United States of America; 3 Department of Behavioral Sciences, Rush University Medical Center, Chicago, IL, United States of America; 4 Department of Neurological Sciences, Rush University Medical Center, Chicago, IL, United States of America; 5 Department of Mental Health, VA Long Beach Healthcare System, Long Beach, CA, United States of America

## Abstract

Risk aversion and temporal discounting are preferences that are strongly linked to sub-optimal financial and health decision making ability. Prior studies have shown they differ by age and cognitive ability, but it remains unclear whether differences are due to age-related cognitive decline or lower cognitive abilities over the life span. We tested the hypothesis that cognitive decline is associated with higher risk aversion and temporal discounting in 455 older persons without dementia from the Memory and Aging Project, a longitudinal cohort study of aging in Chicago. All underwent repeated annual cognitive evaluations using a detailed battery including 19 tests. Risk aversion was measured using standard behavioral economics questions: participants were asked to choose between a certain monetary payment versus a gamble in which they could gain more or nothing; potential gamble gains varied across questions. Temporal discounting: participants were asked to choose between an immediate, smaller payment and a delayed, larger one; two sets of questions addressed small and large stakes based on payment amount. Regression analyses were used to examine whether prior rate of cognitive decline predicted level of risk aversion and temporal discounting, controlling for age, sex, and education. Over an average of 5.5 (SD=2.9) years, cognition declined at an average of 0.016 units per year (SD=0.03). More rapid cognitive decline predicted higher levels of risk aversion (p=0.002) and temporal discounting (small stakes: p=0.01, high stakes: p=0.006). Further, associations between cognitive decline and risk aversion (p=0.015) and large stakes temporal discounting (p=0.026) persisted in analyses restricted to persons without any cognitive impairment (i.e., no dementia or mild cognitive impairment); the association of cognitive decline and small stakes temporal discounting was no longer statistically significant (p=0.078). These findings are consistent with the hypothesis that subtle age-related changes in cognition can detrimentally affect individual preferences that are critical for maintaining health and well being.

## Introduction

Decision making is heavily influenced by individual preferences, in particular those related to taking risks and delaying rewards. Risk aversion, the tendency to prefer a certain (i.e., safe) outcome over an uncertain but potentially greater outcome, and temporal discounting, the tendency to prefer an immediate reward over a delayed but larger reward, have been extensively examined in behavioral economics, neuroeconomics, and psychology and are associated with suboptimal decision making in critical domains such as finance and health [1–14]. For example, highly risk averse individuals often choose safe but low yield investment options, stay in positions of employment with high stability but limited opportunities for advancement [1, 8], and display worse financial and healthcare decision making ability [15]. Similarly, persons who tend to discount future rewards make poorer investment and savings decisions [10, 11], have more debt, are less likely to utilize health insurance, exercise less, and engage in risky behaviors such as unsafe sex [12–14].

Despite the recognition of the importance of individual preferences for decision making, relatively little is known about these preferences in older adults. Prior studies have demonstrated age-differences in risk aversion and temporal discounting [16–18], but the reasons for these differences remain unknown. This reflects an important gap in knowledge, given that older adults face some of life’s most crucial financial and healthcare decisions, many of which require an adequate grasp of risks and time horizons (e.g., estate planning, management of multiple prescriptions, and planning for the end of life). We previously reported that lower cognitive function in late life is associated with higher risk aversion [19] and temporal discounting [20] cross-sectionally. However, work in younger persons has shown that worse educational testing performance is correlated with higher risk aversion [21, 22]and temporal discounting [23–27] earlier in life. Therefore, it remains unclear whether higher risk aversion and temporal discounting in older adults is a result of age-related cognitive decline or, rather, a manifestation of lower cognitive abilities over the life span. To our knowledge, no research has been conducted on the longitudinal association of change in cognitive function with risk aversion and temporal discounting.

In this study, we tested the hypothesis that cognitive decline predicted higher risk aversion and temporal discounting in community-dwelling older adults. Data came from 455 participants without dementia in the Rush Memory and Aging Project [28]. All participants underwent detailed cognitive testing annually and participated in a decision making assessment survey, which included standard behavioral economics questions used to test risk aversion and temporal discounting. We examined the association of the rate of change in global cognitive function in years prior to the decision making assessment with level of risk aversion and temporal discounting in mixed effects models adjusted for age, sex, education. We then excluded persons with mild cognitive impairment (MCI) at the time of decision making assessment to examine the relationship in persons without any overt cognitive impairment (i.e., no dementia or MCI).

## Materials and Methods

### Participants

Participants were enrolled in the Rush Memory and Aging Project, which began in 1997 and is an ongoing longitudinal cohort study of chronic conditions of aging [29] that recruits from around 40 retirement and subsidized housing facilities around the Chicago metropolitan area. All participants signed an informed consent agreeing to annual clinical evaluation. The study was approved by the institutional review board of Rush University Medical Center. Annual clinical evaluation included medical history, neurological and neuropsychological examinations as previously described in detail [29]. In 2010 a decision making assessment which included questions on risk aversion and temporal discounting was added as part of a substudy that was approved by the Institutional Review Board of Rush University following informed consent.

As of November, 2013, 1,671 participants had a complete baseline evaluation for the parent study, of which 83 withdrew from the parent study and 564 died before decision making assessment, and 98 were deemed ineligible for decision making assessment due to cognitive, vision, hearing, or language problems. Of the 926 eligible participants, 802 completed the decision making assessment. Of these, the following were excluded from analyses: 41 who received a diagnosis of dementia at the time of decision making assessment, 7 who had missing temporal discounting or risk aversion measures, and 299 who had only one valid cognitive evaluation at the time of analysis. As a result, data for 455 participants were used in these analyses.

### Cognitive evaluation and clinical diagnosis

Cognitive function was assessed annually via a battery of 21 standard neurobehavioral tests, including the Mini Mental State Examination. Scores on 19 tests were used to create a summary measure of global cognitive function as previously described [28, 30]. To compute the summary measure, raw scores on each of the individual tests within each domain (all 19 for global cognitive function) were converted to z-scores using the baseline mean and standard deviation of the entire cohort, and the z-scores of the tests were averaged [31].

Clinical diagnoses of dementia were conducted at each annual evaluation using a three stage process including computer scoring of neurobehavioral tests, clinical judgment by an experienced neuropsychologist, and diagnostic classification by an experienced clinician, as previously described [29]. Diagnosis of dementia and probable AD followed the criteria of the joint working group of the National Institute of Neurologic and Communicative Disorders and Stroke and the Alzheimer’s Disease and Related Disorders Association [32]. A diagnosis of MCI was rendered for individuals found to have cognitive impairment by the neuropsychologist but who did not meet criteria for dementia [33].

### Assessment of risk aversion and temporal discounting

Risk aversion was assessed via a series of 10 questions used in standard behavioral economics approaches as previously described [19, 34]. Participants were asked, “Would you prefer $15 for sure, OR a coin toss in which you will get $[potential gain greater than $15] if you flip heads or nothing if you flip tails?” Potential gamble gains ranged from $20.00 to $300.00. The gain amounts varied randomly across the series of questions. When the potential gamble is $30.00, the safe payment and the gamble both have the same long run average or expected value. When the potential gamble gain exceeds $30, the expected value of the gamble exceeds the value of the safe payment. Subject specific risk aversion coefficient γ*_i_* was estimated from these 10 questions, and details of the derivation are presented in the statistical analysis.

Temporal discounting was assessed via two sets of questions, one set involving small stakes and one involving large stakes. Small stakes temporal discounting involved 7 binary questions, following a standard preference elicitation protocol [20, 35, 36]. Participants were asked to choose between an immediate smaller payment versus a delayed larger payment, e.g., “Which do you prefer, that you get $10 in cash right now or $14 in a month?” The current payment was fixed at $10 and the delay period was fixed at one month for all questions. Delayed payments ranged from $14 to $30, with payment amounts varying across questions (i.e., they did not escalate in sequence). The Cronbach’s alpha for this measure was 0.87, indicating adequate reliability. Large stakes temporal discounting was assessed via 5 binary questions [9, 10, 35–37] e.g., ‘‘Which do you prefer, that you get $1100 in cash right now or $3000 in a year?” The current payment was fixed at $1000 and the delay period was fixed at one year for all questions. Delayed payments ranged from $1100 to $3000, with payment amounts varying across questions. The Cronbach’s alpha for this measure was 0.81, indicating adequate reliability.

### Demographics

Age was based on self-reported date of birth and date of baseline assessment. Self-reported sex, and education (years of schooling) were recorded at baseline.

### Statistical analysis

Risk aversion coefficient: We estimated the risk aversion coefficient using a well-established behavioral economics approach based on participants’ responses to the 10 risk aversion questions [2, 38, 39] as described previously [15, 19, 34]. The questions involve binary choices between a gamble and a safe payoff. For participant *i* with a risk aversion coefficient *γ*_*i*_, the expected utility of the gamble at the *j*th question, $U_{ij}^{G}$, is defined by the following function:

$$U_{ij}^{G}=0.5\times \frac{Gain_{j}{}^{1-\gamma_{i}}}{1-\gamma_{i}}$$

where *Gain*_*j*_ is the winning outcome in the *j*th gamble, while the safe option payoff for *i*th participant at *j*th question has the expected utility:

$$U_{ij}^{S}=\frac{Safe_{j}{}^{1-\gamma_{i}}}{1-\gamma_{i}}$$

where *Safe*_*j*_ is the safe gain for the *j*th question. If observed outcomes in the trials are *Y* and the decision of choosing the gamble is *Y = 1*, the probability *P(Y = 1)* depends on the difference between expected utility of the gamble and safe option. Therefore, the odds of choosing the gamble over safe option were formulated as:

$$\frac{P(Y=1)}{1-P(Y=1)}=e^{U_{ij}^{G}-U_{ij}^{S}}$$

A positive $U_{ij}^{G}-U_{ij}^{S}$ suggests that a participant favored the gamble (i.e., odds greater than 1). The subject-specific risk aversion coefficient γ_i_ was estimated using the above formula.

Temporal discounting: We estimated the subject specific discounting rate *α* using a well-established hyperbolic function [9, 37, 40, 41] and described previously in our cohort [20, 42, 43]:

$$V=\frac{A}{1+\alpha D}$$

where *V* represents the discounted value of the delayed reward *A* at delay *D*. The function shows that larger discounting rates (*α*) correspond to smaller values of *V*. The odds of choosing the delayed reward over the immediate reward were formulated as:

$$\frac{P(Y=1)}{P(Y=0)}=e^{V-C}$$

where the observed outcome of a trial is denoted by *Y*, the decision to choose the delayed reward by *Y = 1* and the decision to choose the immediate reward by *Y = 0*. The probability of choosing the delayed reward, *P(Y = 1)*, depends on the difference between the discounted delayed reward *V* and the immediate reward *C*. If *V—C* is positive, this indicates a preference for the delayed reward with odds greater than 1, and a negative *V—C* indicates a preference for the immediate reward. The discounting rate α for both large stakes and small stakes questions could be estimated from the above equations.

After computing subject specific risk aversion and temporal discounting statistics, we examined the bivariate associations of these statistics with demographics and relevant covariates. Then, to investigate the temporal association between the prior rate of change in cognition and risk aversion and temporal discounting, we first estimated the slope of cognitive decline for each individual. To do this, we fit a general linear mixed model to longitudinal cognitive testing data up until the time of first assessment of risk aversion and temporal discounting, adjusted for baseline age, gender, and years of education. These person-specific slopes were then used in nonlinear mixed effect models as the predictor of risk aversion and temporal discounting. The model details were published previously [19, 20]. In these models, a negative coefficient indicates that a greater rate of cognitive decline (i.e., a more negative slope for change in cognition) is related to a higher level of the outcome of interest. All models were adjusted for age, sex, and education. In the final step, we repeated analyses after excluding persons with mild cognitive impairment (MCI). Programming was done in SAS 9.2 and models were validated graphically and analytically [44].

## Results

### Descriptive properties of sample

Participants without dementia (n = 455) had a mean age of 83.6 years (SD = 7.5) and mean education of 15.2 (SD = 3.0) years, 75.6% were female, and 89.2% were white, non-Hispanic. The average MMSE score at baseline was 28.6 (SD = 1.5). An average of 5.5 (SD = 2.9, range: 0.4–14.8) years of annually assessed data on cognitive function were available. The mean level of global cognitive function at baseline was 0.29 standard unit (SD = 0.45, range: -1.18–1.39). Global cognition declined at an average of 0.018 units per year (SD = 0.03, range: -0.19–0.07).

### Descriptive properties of risk aversion and temporal discounting

The mean estimate of risk aversion γ derived from participants’ responses to all risk aversion questions was 0.37 (SD = 0.30; range, 0.07–0.90), with higher values indicating greater risk aversion. Risk aversion was negatively correlated with global cognition (r = -0.12, p = 0.009) and male sex (r = -0.14, p = 0.01) such that persons with lower cognitive function and women exhibited more risk aversion on average.

The mean discount rate α was 0.018 (SD = 0.023, range: 0.002–0.087) for small stakes and 0.67 (SD = 0.84, range: 0.06–2.78) for large stakes, with larger values indicating greater discounting. Small stakes temporal discounting was negatively correlated with global cognition (r = -0.14, p = 0.003), education (r = -0.14, p = 0.004), and male sex (r = -0.13, p = 0.005), such that persons with lower cognitive function, lower education, and women exhibited more discounting on average. Large stakes temporal discounting was negatively correlated with baseline global cognition (r = -0.12, 0 = 0.014) and male sex (r = -0.12, p = 0.01), such that persons with lower cognitive function and women exhibited more discounting on average at large stakes.

Risk aversion was weakly correlated with both large (r_spearman_ = 0.23, p<0.001) and small stakes temporal discounting (r_spearman_ = 0.26, p<0.001). Large and small stakes temporal discounting were more strongly correlated with each other (r_spearman_ = 0.61, p<0.001).

### Relation of cognitive decline with risk aversion and temporal discounting

**Fig 1:**
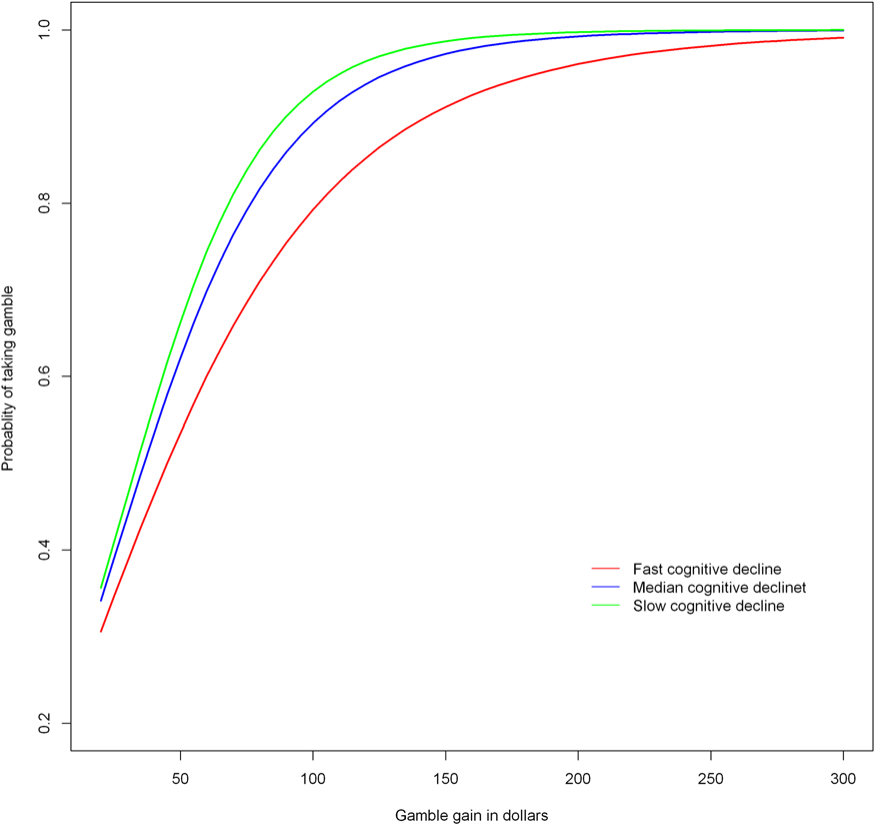
Association of cognitive decline with risk aversion as derived from a non-linear mixed effects model. The figure depicts the probability of taking the gamble as a function of the gamble gain in dollars. Lower curves on the Y axis indicate more risk aversion. Predicted curves are shown for a typical participant (i.e., female with median age, education, and income) at three different levels of cognitive decline: red indicates fast (highest 10^th^ percentile), blue indicates median, and green indicates slow (lowest 10^th^ percentile) cognitive decline.

To test the hypothesis that the prior rate of change in global cognition predicted the level of risk aversion, we constructed a nonlinear mixed effect model with risk aversion as the outcome and terms for global cognitive slope (i.e., annual rate of cognitive change), age at risk aversion assessment, sex, and education. The results of this analysis showed that a more rapid rate of cognitive decline was associated with higher risk aversion (Table 1, Fig. 1). To clarify this effect, when the rate of decline in global cognition increased by 1 standard deviation (of the slope), the risk aversion score was on average 45% higher than the mean risk aversion score.

**Table 1:** Risk aversion and temporal discounting as a function of cognitive decline.

Outcome:	Risk aversion		Temporal discounting, small stakes		Temporal discounting, large stakes	
	Est (SE)	p	Est (SE)	p	Est (SE)	p
Age	-0.01 (0.02)	0.78	-0.01 (0.01)	0.58	0.01 (0.01)	0.81
Male	-1.08 (0.47)	0.022	-0.56 (0.20)	0.005	-0.58 (0.24)	0.015
Education	-0.03 (0.06)	0.62	-0.07 (0.03)	0.009	-0.07 (0.03)	0.035
Cognitive decline	-15.50 (4.86)	0.002	-6.81 (2.67)	0.011	-8.84 (3.23)	0.006

**Fig 2:**
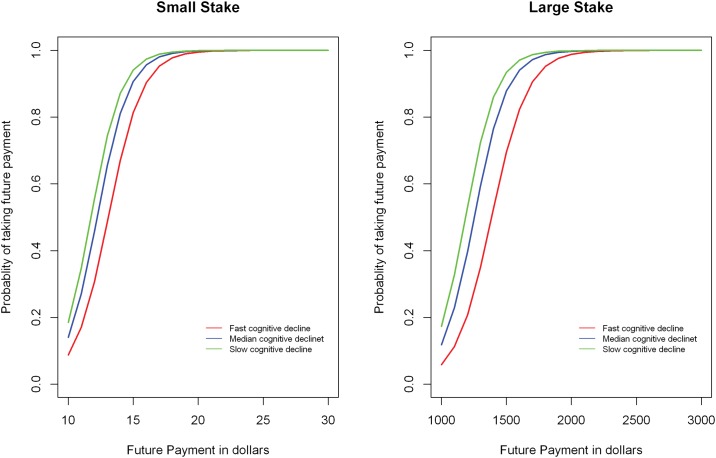
Association of cognitive decline with temporal discounting (small and large stakes) as derived from a non-linear mixed effects model. The figure depicts the probability of taking future payment rather than a fixed immediate payment as a function of the future payment in dollars. Lower curves on the Y axis indicate more temporal discounting. Predicted curves are shown for a typical participant (i.e., female with median age, education, and income) at three different levels of cognitive decline: red indicates fast (highest 10^th^ percentile), blue indicates median, and low indicates slow (lowest 10^th^ percentile) cognitive decline.

A more rapid rate of cognitive decline was also associated with higher levels of temporal discounting, for both small and large stakes (Table 1, Fig. 2). To clarify this effect, when the rate of decline in global cognition increased by 1 standard deviation (of the slope), the small stakes temporal discounting score was on average 24% higher than the mean score, and the large stakes temporal discounting score was on average 32% higher than the mean score.

### Relation of cognitive decline with risk aversion and temporal discounting after excluding MCI

To ensure that the association of cognitive decline with risk aversion was not driven by persons at the lower end of cognitive ability, we conducted sensitivity analyses in which we repeated the model after excluding persons with MCI (n = 97, 21.3% of analytic cohort). The association of cognitive decline with risk aversion was marginally attenuated after exclusion of persons with MCI but remained significant (estimate = -12.28, SE = 5.03, p = 0.015). The association of cognitive decline with temporal discounting was marginally attenuated but remained significant for large stakes (estimate = -9.44, SE = 4.23, p = 0.026). By contrast, it was marginally attenuated and no longer significant for small stakes (estimate = -4.97, SE = 2.81, p = 0.078).

## Discussion

We examined the association of cognitive decline with two well-established individual preferences, risk aversion and temporal discounting, in a cohort of more than 450 community-dwelling older men and women who did not have dementia. We found that, after adjusting for differences in age, sex, and education, higher rates of cognitive decline in preceding years was related to greater risk aversion and temporal discounting. Subsequent analyses showed that rate of cognitive decline was associated with these preferences in older persons without any evidence of cognitive impairment. This study is the first that we are aware of to demonstrate that declines in cognitive abilities are related to these preferences in old age. Overall, these findings are consistent with the hypothesis that the subtle cognitive decline observed in older adults considered cognitively normal negatively affects individual preferences that are strongly linked to important financial and health decisions.

In prior work, age-differences in risk aversion and temporal discounting have been reported [16–18] and we previously reported cross-sectional associations of cognitive function with risk aversion [19] and temporal discounting [20] in older persons. However, it is difficult to discern from cross-sectional data whether the association of cognition with these preferences in older persons was longstanding (reflecting a level effect). Research in younger persons has shown that higher IQ and performance on educational attainment tests are negative correlates of risk aversion and temporal discounting [21–25, 45]; thus cross-sectional associations in later life may simply indicate that older persons with lower cognitive abilities were more risk averse and more likely to temporally discount throughout their entire lives. This study extends prior work by demonstrating that change in cognitive function, rather than just level of function, predicts risk aversion and temporal discounting preferences. This is important because it suggests that age-related cognitive decline may lead to older adults being more risk averse and more likely to discount future rewards. The longitudinal findings imply that older persons who are beginning to experience subtle cognitive declines may begin to lose the ability to optimally weigh preferences involving gambles or foresight into the future, and thus may defer to the “safe” or “immediate” option to the detriment of their well-being in the long-term. Further research is needed to establish that older adults exhibit changes in their level of risk aversion and temporal discounting in response to declining cognition.

These findings could have important implications for the health, well-being, and financial security of older adults. Older adults are faced with many complex and consequential decisions at a time in their lives when, paradoxically, many of them begin to experience declines in their cognitive abilities. Findings such as these could indicate that age-related changes in cognition that are often considered benign can have negative ramifications. Research has shown that many cognitively intact older adults make sub-optimal choices regarding health and finances compared to younger persons [16, 46–49], and fraud and other forms of exploitation are major problems among older persons [50, 51]. Though more research is needed to further understand the complex sources of these age-related differences, part of the reason for this may be the inability to navigate risk/benefit ratios of trade-offs between instant gratification and future payoffs. Risk aversion [1–7] and temporal discounting [9–11, 35, 52] have been shown to be associated with numerous detrimental real world economic and health behaviors and outcomes, and we have shown that risk aversion is associated with poorer health and financial decision making [15] and that temporal discounting is associated with increased mortality [42] in this cohort. These data are consistent with the notion that subtle, preclinical age-related cognitive decline may have important functional consequences including altered preferences and, ultimately, impaired decision making [53, 54]. However, more research is needed to investigate whether these preferences actually change in later life.

Although the neurobiological substrate underlying the association between cognitive deterioration and increased risk aversion and temporal discounting remains unclear, our group has shown differences in neural intrinsic connectivity networks among older persons according to risk aversion preference using resting-state functional magnetic resonance imaging [34]. Connectivity of ventral frontal regions implicated in fear processing was associated with greater risk aversion, while connectivity of dorsal frontal regions implicated in logical reasoning was associated with less risk aversion. We have also demonstrated different functional connectivity patterns associated with level of temporal discounting in this cohort [43]. Connectivity of ventromedial prefrontal regions implicated in poor impulse control was associated with greater temporal discounting, while connectivity of parahippocampal regions implicated in memory was associated with less temporal discounting. Research is needed to determine if the differences in functional network characteristics related to higher risk aversion and temporal discounting are a feature of age-related cognitive deterioration, and if they are associated with common neuropathologies that contribute to dementia such as Alzheimer’s disease and cerebrovascular disease.

Strengths of this study include the robust evaluation of risk aversion and temporal discounting, as well as the use of longitudinal data on cognitive function from annual assessments using a comprehensive battery of neuropsychological tests in a relatively large cohort of community-based older persons free of dementia. The study adjusted for demographic factors and tried to ensure that the findings were not due to the inclusion of persons with preclinical cognitive impairment using sensitivity analyses. A limitation of the study is the selected nature of this volunteer cohort, which may limit the generalizability of our findings. Another limitation is the use of hypothetical questions regarding monetary payouts rather than involving actual monetary payouts, although a number of prior studies using similar hypothetical tasks to those used here have established that risk aversion and discounting tendencies are associated with psychological problems, and poorer occupational and financial outcomes [9, 10, 35]. Finally, although cognitive function was assessed longitudinally in this study, risk aversion and temporal discounting were only assessed at one time point; therefore, actual changes in these preferences in response to cognitive decline could not be assessed. The alternate hypothesis that risk aversion and temporal discounting are stable, trait-like characteristics that predict late-life cognitive decline could not be assessed. Similarly, we could not assess the alternate hypothesis that these preferences do change over time, but such changes precede and predict cognitive decline. However, risk aversion and temporal discounting are assessed annually in this cohort; therefore, future studies with multiple waves of preference assessments will be able to address these alternate hypotheses.

This is the first longitudinal study that we are aware of to establish the relationship of cognitive decline with risk aversion and temporal discounting in cognitively non-impaired older persons. Given that there are 40 million persons above age 65 in the U.S. and this number is growing rapidly, and this group holds the majority of the nation’s wealth as well as health burden, these findings could have important ramifications to our society as a whole. These findings could suggest that older persons who are beginning to experience subtle declines in their cognitive abilities may benefit from assistance in understanding and appreciating risk/benefit ratios and time trade-offs. Future longitudinal studies are needed to show whether declining cognition leads to changes—specifically, increases—in risk aversion and discounting during the aging process.
